# Strategies to improve postpartum engagement in healthcare after high-risk conditions diagnosed in pregnancy: a narrative review

**DOI:** 10.1007/s00404-024-07562-7

**Published:** 2024-05-24

**Authors:** Naomi C. A. Whyler, Sushena Krishnaswamy, Sarah Price, Michelle L. Giles

**Affiliations:** 1 Department of Obstetrics and Gynaecology, Monash University, Wellington Road, Clayton, VIC 3800 Australia; 2 Department of Medicine, Royal Melbourne Hospital, University of Melbourne, Royal Parade, Parkville, VIC 3000 Australia; 3 Department of Obstetric Medicine, Royal Women’s Hospital, 20 Flemington Road, Parkville, VIC 3000 Australia; 4 Department of Infectious Diseases, University of Melbourne, Grattan Street, Parkville, VIC 3000 Australia

**Keywords:** Postpartum period, Diabetes, Gestational, Hypertension, Pregnancy-induced, Pre-eclampsia, Healthcare systems

## Abstract

Transition from antepartum to postpartum care is important, but often fragmented, and attendance at postpartum visits can be poor. Access to care is especially important for individuals diagnosed antepartum with conditions associated with longer-term implications, including gestational diabetes (GDM) and hypertensive disorders in pregnancy (HDP). Strategies to link and strengthen this transition are essential to support people to attend recommended appointments and testing. This narrative review evaluates what is known about postpartum transition of care after higher-risk antepartum conditions, discusses barriers and facilitators to uptake of recommended testing, and outlines strategies trialled to increase both postpartum attendance and testing. Barriers to attendance frequently overlap with general barriers to accessing healthcare. Specific postpartum challenges include difficulties with transport, coordinating breastfeeding and childcare access. Systemic challenges include inadequate communication to women around implications of health conditions diagnosed in pregnancy, and the importance of postpartum follow up. Uptake of recommended testing after a diagnosis of GDM and HDP is variable but generally suboptimal. Strategies which demonstrate promise include the use of patient navigators, focused education and specialised clinics. Reminder systems have had variable impact. Telehealth and technology are under-utilised in this field but offer promising options particularly with the expansion of virtual healthcare into routine maternity care. Strategies to improve both attendance rates and uptake of testing must be designed to address disparities in healthcare access and tailored to the needs of the community. This review provides a starting point to develop such strategies from the community level to the population level.

## Current recommendations for postpartum care

The postpartum period begins at birth and is defined as the first 6 [1] to 12 [2] weeks after delivery, sometimes referred to as the fourth trimester. Postpartum review with a healthcare practitioner plays a significant role in supporting those with immediate health needs including contraception, breastfeeding, peripartum mental health, and wound care [1, 3]. Most guidelines recommend this visit takes place at or prior to 6 weeks’ postpartum [1].

Despite this recommendation, documented low attendance rates at the postpartum visit suggest that many people do not receive this important review in a timely manner [4]. Contributing factors to this include inconsistently defined care pathways supporting people in the transition from antepartum to postpartum care, fragmented communication between healthcare providers and competing priorities.

Many pregnant people are diagnosed for the first time with a medical condition during pregnancy. Conditions such as gestational diabetes (GDM) and hypertensive disorders of pregnancy (HDP) have implications for longer term health and ongoing engagement in healthcare and postpartum testing is recommended following these diagnoses. GDM, defined as the first presentation of hyperglycaemia in pregnancy, affects 14% of pregnancies worldwide [5] and is predictive of an increased risk of metabolic disorders including a seven- to ten-fold risk of type 2 diabetes within 10 years [6]. An oral glucose tolerance test (OGTT) is recommended for those with a diagnosis of GDM within the first 12 weeks’ postpartum [2] and at regular intervals thereafter to provide opportunity for early diagnosis and treatment of type 2 diabetes mellitus.

The spectrum of HDP includes gestational hypertension, pre-eclampsia, eclampsia, chronic hypertension and pre-eclampsia superimposed on chronic hypertension [7]. Global prevalence is estimated at 116 cases per 100,000 women of childbearing age, with the highest rates seen in low-income countries at 286 cases per 100,000 compared with 70 cases per 100,000 in high-income countries [8]. HDP are associated with long-term cardiovascular, cerebrovascular and renal disease [9, 10], and blood pressure review is recommended in the first 7–10 days postpartum [7]. Testing for persistent hypertension, markers of organ dysfunction (e.g. urinalysis for proteinuria, renal and liver function testing), ambulatory blood pressure monitoring, metabolic screening with fasting glucose, lipid profile and body mass index measurement, within 3–6 months is advised, and at regular intervals thereafter [11].

Both chronic medical illness [12] and new antepartum diagnoses of medical conditions [13] are associated with poorer peripartum mental health. Those with GDM have a significantly increased risk of postpartum depressive symptoms, with an estimated pooled relative risk of 1.32 [14]. Pre-eclampsia has been identified as a risk factor for depression both during the postpartum period, and beyond [15]. The relationship between other HDP and poor peripartum mental health is less clear, but trends towards increased risk of depression, anxiety and post-traumatic stress disorder have been reported [16]. Postpartum review provides an important opportunity for screening and support for peripartum mental health disorders.

This state of the science review summarises what is known about rates of postpartum engagement in care both nationally and internationally, as defined by attendance at the 6-week postpartum visit, and uptake of recommended testing in those with medical diagnoses of GDM and HDP during pregnancy. We outline the strategies trialled to date to improve attendance at postpartum visits and improve adherence to recommended testing; the barriers and facilitators to uptake of recommended testing; and discuss gaps in the literature for future research.

## Attendance postpartum: what is known?

Attendance rates for postpartum care vary significantly between different cohorts, countries, and healthcare systems. Whilst a 6-week appointment is recommended, few studies report attendance rates at this time point. Attendance rates of 42% at the maternal 6-week check have been reported in the United Kingdom (U.K.) by 6–8 weeks postpartum, increasing to 62% by 12 weeks [17]. In the United States (U.S.), reported rates vary from 54% in the first year postpartum amongst veterans [18], to 72% in a recent systematic review [4], and to more than 80% by 90 days postpartum amongst women with funded postpartum care [19]. In Australia, 81% attendance at a general practitioner visit was reported in a South Australian cohort in the first 9 months postpartum [20]. Disparities in attendance rates exist within these countries, with significantly lower attendance rates in the U.S. among those of Black/African American (52%), Asian (45%) and Hawaiian/other Pacific Islander (48%) race and ethnicity [18]. Similarly, in Australia attendance was lower (48%) amongst Aboriginal and Torres Strait Islander people attending rural health services [21], suggesting multiple factors contributing to non-attendance. Access to health insurance in countries such as the U.S. also impact on attendance at postpartum visits, with Medicaid-insured women less likely to attend than those with private insurance [4]. Concurrent existence of a chronic condition has also been found to reduce the likelihood of attending the postpartum visit [4].

## What strategies have been trialled to increase postpartum attendance?

A variety of strategies have been trialled in different settings to increase postpartum attendance. The different types of approaches are discussed by Phillips et al. [22] in their recent review of postpartum transition of care, and strategies trialled to date were described by Stumbras et al. [23] in their 2016 narrative review. As these summaries provide an excellent overview of strategies to improve postpartum transition of care, an in-depth analysis is not repeated in our review. For the purposes of understanding what has been identified to date, Table 1 summarises these strategies with examples of their use to increase postpartum attendance.

**Table 1:** Strategies to increase postpartum engagement, with examples of use

Strategy	Detailed description	Examples of use
Administrative assistance	Pre-discharge scheduling of postpartum appointments	A retrospective study in Lebanon found that being given an appointment for postpartum follow-up before discharge increased attendance rates compared with those who were not [24]
Patient education	Provision of written pamphlets Online resources with patient-facing documents including guideline summaries and postpartum visit guides Antepartum group and individual education sessions	4th Trimester Project™: website providing resources for new mothers such as ‘Postpartum toolkit’ to plan visits, embedded videos on health warning signs and linking users with each other for support [24]
Home visit	Nurse, midwife or doula-led home visits	Nurse-Family Partnership [26] (Ontario, Canada): nurse-led bi-weekly home visits for young, first-time mothers on low income during pregnancy and to 2 years’ postpartum
Patient incentives	Provision of coupon or infant-related items upon attendance at appointment	Incentivised postpartum care [27] (Colorado, U.S.): provision of infant carrier to adolescents who returned for postpartum check within 12 weeks
Patient care co-ordinator	Healthcare practitioner with administrative focus in scheduling appointments and identifying those who need additional assistance with rescheduling appointments and tests	The Diabetes in Pregnancy Program (Houston, U.S.) used patient care co-ordinators to arrange postpartum OGTT testing and help with overcoming barriers to attendance [28]
Patient navigator	Trained individuals who help with navigating the healthcare system, often incorporating culture-specific approaches over a longitudinal time course. Input includes booking interpreters, organising transport, filling in forms	Familias Sanas [29] (Phoenix, U.S.): trained social work students helped Latina/Hispanic women communicate with healthcare providers and develop antepartum and postpartum visit plan
Reminder system	Use of a letter, email, text, telephone-based or other messaging system to remind individuals or their healthcare providers to attend for review or for testing	Healthy Beyond Pregnancy [30] (Pittsburgh, U.S.): after scheduling a postpartum visit, ‘nudge’ text messages sent within 48 h of hospital discharge, and within 72 h of scheduled visit
Specialist postpartum clinic	Provision of a specialist clinic with the primary aim of providing postpartum care with access to a team of transdisciplinary care providers	Two-Generation Clinic [31] (Chicago, U.S.): co-scheduled, co-located dyad clinic visits set up within combined Internal Medicine/Paediatric settings, with primary care provider and in-clinic access to nursing, social work, lactation, psychiatrist and obstetrician-gynecologist support
Telehealth	Use of text, mobile application or web-based technology. Often used in tandem with other strategies e.g. education, reminder system	Pilot mHealth programme (New Jersey, U.S.): text-based two-way messaging system for 6 weeks postpartum with daily six-item questionnaire. ‘Yes’ responses triggered telephone follow-up by provider [32]

*OGTT* oral glucose tolerance test, *U.S.* United States

Patient incentives, patient education and home visits have generally shown mixed success at increasing attendance. Patient navigator systems appear more successful at increasing attendance as well as other parameters including vaccination uptake and screening for depression [33]. This may be due to the ability to tailor such strategies towards individual needs. A systematic review and meta-analysis by Bowden et al. [34] of strategies in high-income countries and their impact on antepartum and postpartum care found that people who received enhanced support with individualised care were significantly more likely to attend their postpartum visit. This conclusion was based on aggregate data using two very different strategies: patient incentives [27] and patient navigators [29]. The use of administrative assistance such as pre-discharge appointment scheduling has also been associated with promising results [24] but has yet to be trialled prospectively as a strategy in its own right.

More recently, the use of technology has allowed the opportunity to expand strategies beyond reminder-based messaging alone, for example to incorporate remote blood pressure monitoring [35], and education delivery [32]. Use of technology within the postpartum setting to specifically increase attendance rates has yet to be explored definitively particularly in high-income settings. Preliminary findings from a series of qualitative interviews for people who received text-based messaging from their postpartum healthcare provider indicated that this approach was viewed positively by participants [32].

Many programmes combine two or more of these approaches within their postpartum care set-up. For example, Health Beyond Pregnancy [30] used text-based reminder systems to prompt postpartum follow-up, with additional motivational messaging via video and text, and also used a cash incentive which was provided to those who attended their visit. This multi-approach focus may be helpful in broadening the reach of individual programmes; however, there is insufficient evidence to identify which combinations of approaches are most likely to be successful in increasing attendance rates. Feasibility assessment and economic evaluation of these combined approaches are also an essential part of service evaluation and data remains sparse on this topic.

## Uptake of postpartum testing: what is known?

Recommended postpartum testing for those with GDM includes an OGTT within the first 12 weeks [2], and for those with HDP, a blood pressure measurement within 7–10 days [7], followed by more comprehensive testing with proteinuria analysis, ambulatory blood pressure testing and metabolic screening [11]. However, postpartum testing rates after antepartum diagnoses such as GDM and HDP are frequently suboptimal.

A systematic review of studies analysing postpartum testing rates after GDM identified aggregate rates of 48% [36], with a further systematic review focusing on studies published in the U.S. setting since 2011 finding testing rates of 58% at best, and frequently lower, by 3 years postpartum [37]. Analysis from a large university-insurance collaborative data repository in the U.S. identified much lower testing rates after GDM diagnosis with just 6% undergoing any type of glucose testing by 8 weeks postpartum, 22% by 1 year and 51% by 3 years [38]. Blood pressure screening within 6 weeks of delivery after HDP also varies, with observational studies from the U.S., reporting ranges from 33.5% [39], to 52% [40]. A further evaluation of women attending a 3-month study visit found that despite high rates of blood pressure testing (98%) in this group, far fewer (< 60%) had further testing with lipid screening [41]. National data in other high-income settings is sparse.

In Australia, those diagnosed with diabetes are encouraged and assisted to register with the National Diabetes Services Scheme (NDSS); registrants are invited to being contacted for research purposes using an opt-in model of consent [42]. A survey of responses among those registered to the NDSS with GDM identified that 73% underwent postpartum diabetes testing, but of these, only 27% underwent OGTT prior to 8 weeks’ postpartum [42]. Lower rates were reported in more remote regions of Australia, with 14% of Indigenous people, and 28% of non-Indigenous people undergoing OGTT by 6 months postpartum [43]. Health inequities in this study were identified beyond testing rates, with Indigenous people experiencing significantly longer times to any postpartum glucose test [43]. A large retrospective study reviewed the follow up of over 10,000 women with GDM from an estimated 12 weeks’ postpartum, until over 4 years postpartum, and found that 29% had not been assessed for diabetes [44]. This study excluded the postpartum period in order to highlight practices of long-term screening, finding that longer term follow-up also remains suboptimal [44]. The authors also found that 6% of the whole cohort received a new diagnosis of diabetes within this timeframe, highlighting the importance of testing in the postpartum period and beyond [44]. In terms of follow up of HDP in the postpartum period, 94.5% of a group of women with diagnosis of pre-eclampsia in the previous 2 years reported blood pressure measurement since birth, but screening for metabolic abnormalities occurred in less than half [45].

## Factors associated with adherence to recommended testing

In order to identify why some strategies work better than others, it is essential to understand the facilitators and barriers for uptake of testing. Several individual, sociodemographic and systemic factors contribute to the ability and prioritisation of engagement in care (Table 2).

**Table 2:** Factors associated with uptake of testing and non-uptake of postpartum testing after high-risk conditions GDM and HDP

	Factors associated with non-uptake of testing	Factors associated with uptake of testing
Individual: Clinical factors	Mental health disorders in pregnancy [37] Raised body mass index at GDM diagnosis [37, 46] Increased gestational weight gain [28] Past history of GDM [46] Not breastfeeding [47] Readmission to medical care (woman or baby) [48]	Concurrent diagnosis of pre-eclampsia [37]
Individual: Health-seeking behaviours	Time constraints [49, 50] Household emergency [48] Need to breastfeed at testing centre [49] Need to care for other children [48, 50] Busy looking after their infant [50]	Attendance at postpartum visit [36, 51]
Individual: Health literacy and beliefs	Belief that conditions no longer exist postpartum [49] Inadequate healthcare education [49]	Increased awareness of need for testing [49] Higher level of health literacy [41]
Sociodemographic	Higher parity [47] People who smoke [28, 37, 47] Lower education level [37] Residence: rural area [52] or in area of deprivation [47] Unemployed [47] Lack of transport [48, 49] Race and ethnicity: Black individuals [37, 40] in U.S.; individuals of Eastern European and Caribbean race and ethnicity in U.K. [47]; individuals of Indigenous background in Australia [43]	Nulliparity [36, 37] or fewer children [46] Married or partnered [28, 37, 41] Higher education level [36, 51] Private health insurance [37, 41] Not speaking English as primary language [28, 51] Race and ethnicity: Asian [37, 51] and Mexican American [46] participants in U.S
Healthcare provider factors	Lack of communication between healthcare providers [6] Lack of documentation [6]	Reminders and advice from healthcare practitioners [42, 49] Receiving written information after the birth [42] Receiving input from obstetrician and diabetes educator about GDM during pregnancy [42]
Health systems factors	Burdensome test (OGTT) [6] Unpleasant test (needles, need to fast, OGTT drink) [49]	Child-friendly facilities at testing centres [49] Breastfeeding-friendly facilities at testing centres [49] Factors reducing time burden on testing [49, 50]

*GDM* gestational diabetes mellitus, *OGTT* oral glucose tolerance test, *U.K.* United Kingdom, *U.S.* United States

Many of the identified barriers are frequently associated with worse health outcomes across the life course, and often co-exist with each other. For example, people with GDM of high socioeconomic status are more easily able to overcome financial barriers to undergoing oral glucose tolerance testing [53], and those who live rurally [52], or in an area of deprivation [47], are more likely to experience barriers to uptake of testing. Black and Hispanic individuals are over-represented among people with the diagnosis of GDM and HDP [54] yet have lower rates of uptake of postpartum testing [55]. The presence of structural racism within policy and institutional practices may exacerbate such disparities [54]. There is a need to provide tailored, culturally sensitive strategies which factor in social determinants of health.

For many, the logistics of attending for testing present barriers, for example accessibility issues for those requiring childcare and breastfeeding facilities [49], and the time involved in undergoing an OGTT [50]. There is conflicting data on the relationship between undergoing postpartum testing, and factors such as maternal age [28, 36, 37, 40, 42, 47], requirement for insulin for GDM management [36, 46, 47, 56], and the timing of first presentation for antepartum care [37, 51].

## What strategies have been trialled to improve postpartum testing rates in higher-risk groups?

Table 3 presents an overview of strategies trialled within the antepartum and postpartum periods with the aim of increasing postpartum testing after GDM and HDP.

**Table 3:** Interventions to increase attendance at postpartum testing in those diagnosed antepartum with conditions associated with longer-term morbidity

Author	Study design	Target population	Population size	Strategy	Results*
Antepartum setting					
Patient education					
Stasenko et al. 2011 [55]	Retrospective Cohort	People with GDM in San Francisco, U.S	805	Single third-trimester education session with diabetes educator	Increase in postpartum testing (fasting glucose or OGTT) from 33 to 53% (*P* < 0.001)
Multi-approach programme					
Huynh et al. 2016 [57]	Retrospective Cohort	People with GDM in Philadelphia, U.S	292	Intensive diabetes in pregnancy programme, with antenatal education, nutrition visit, booklets, a nurse care co-ordinator to assist with booking appointments, and a reminder to providers situated within the electronic charting system	Increase in postpartum testing from 25 to 49% (odds ratio [OR] 2.98, 95% CI, 1.34 to – 6.62, *P* = 0.007)
Both antepartum and postpartum setting					
Patient care co-ordinator					
Dietz et al. 2008 [51]	Retrospective Cohort	People with GDM in Oregon and Washington, U.S	1332	Care co-ordinator nurse for pregnancy and up to 6-week visit	Increase in postpartum testing from 9 to 58% (*P* < 0.01)
Hunt & Conway 2008 [46]	Prospective Cohort	People with GDM in Texas, U.S	707	Case manager nurse who followed up those who did not undergo OGTT prior to a scheduled postpartum visit, and arranged in-home test if needed	Increase in postpartum glucose screening, (57%) compared to historical rate of 18%
Postpartum setting					
Administrative assistance					
Tsai et al. 2011 [58]	Retrospective Cohort	People attending for postpartum review including GDM in Hawaii, U.S	17	Postpartum appointment provided for people at time of discharge, with incentive of infant photograph taken at first postpartum visit and given to parent at second postpartum visit	No significant difference in rates of postpartum OGTT (5% from 2%, P = 0.155) but small numbers with GDM (17 of total 221)
Patient care co-ordinator					
Mendez-Figueroa et al. 2014 [28]	Retrospective Cohort	People with GDM in New England, U.S	398	Nurse and bilingual worker arranged testing, appointments, reminders and rescheduling for a 4–6 week OGTT	Increase in postpartum testing from 43 to 59% (P < 0.001, hazard ratio 1.59, 95% CI 1.20 – 2.12)
Patient navigator					
Schwartz et al. 2021 [59]	Interventional Cohort Study	People with chronic hepatitis B infection in New York, U.S	1449	Patient navigators provided education on hepatitis B and conducted individual assessments of barriers and facilitators to engagement in care, with subsequent targeted assistance for identified barriers	Increase in postpartum follow-up with hepatitis B specialist at 6 months from 52 to 73% (aOR 1.66, 95% CI 1.39 -1.98)
Reminder system					
Clark et al. 2009 [60]	Randomised Controlled Trial	People with GDM in Ottawa, Canada	223	Postal reminders sent to participants and/or clinicians at 3 months postpartum	Increase in postpartum OGTT testing from 14 to 52% with clinician reminder, 55% with patient-reminder, and 61% with both clinician and patient reminders (*P* < 0.05)
Lega 2012 [61]	Retrospective Cohort	People with GDM in Toronto, Canada	314	Provision of a checklist for physicians to ensure participants had an OGTT pathology form, postpartum appointment, and postpartum education package detailing diabetes screening and prevention	Increase in postpartum OGTT testing from 36 to 62% (*P* < 0.0001, odds ratio [OR] 2.99, 95% CI 1.84 – 4.85)
Shea 2011 [56]	Interventional Cohort Study	People with GDM in Ontario, Canada	262	Three sites were compared: site A provided a postal reminder re: diabetes testing and an OGTT pathology form; site B provided a letter or telephone call and pathology form; a third site, ‘non-reminder site’, received no prompts to remind them about postpartum testing	Increase in OGTT testing within 6 months postpartum from 14% at the non-reminder site, to 23% at site A, and 36% at site B (*P* = 0.01)
Triebwasser et al. 2023 [62]	Randomised Controlled Trial	People with HDP in Pennsylvania, U.S	222	Participants were reviewed in postpartum clinic between four to 12 weeks’ postpartum. A reminder system was trialled: half had an inbox message or ‘nudge’ sent to their obstetric clinician containing clinical details, counselling phrases and contact details for primary care provider. Secondary outcome included attendance at a primary care or specialist visit excluding routine postpartum visit	No difference in attendance at preventive care after the postpartum visit within 6 months was observed (24.6% usual care vs 22.1% intervention; adjusted relative risk [aRR] 0.91, 95% CI 0.57 – 1.47)
Van Ryswyk et al. 2015 [63]	Randomised Controlled Trial	People with GDM in Adelaide, Australia	276	Control group received a single text message reminder at 6 months postpartum, intervention group received a text message reminder at 6 weeks postpartum to undergo an OGTT, followed by further reminders at 3 and 6 months if no notification of testing was received	No difference in testing rates, 77% vs 78% (relative risk [RR] 1.01, 95% CI 0.89 – 1.15)
Specialist postpartum clinic					
Celi et al. 2019 [48]	Retrospective Cohort	People with HDP attending a postpartum cardiometabolic clinic in Boston, U.S	412	Postpartum transition clinic with an Internal medicine specialist to manage hypertension, review additional medical and postpartum issues, and facilitate transition to primary care ongoing	All participants performed home blood pressure monitoring. Attendance at primary care transition appointment was 80%. No pre-intervention data available for comparison
Countouris et al. 2022 [64]	Retrospective Cohort	People attending multidisciplinary postpartum HDP clinic in Pennsylvania, U.S	140	People with HDP seen at a specialist clinic (Materno-Fetal Medicine and Cardiology) attended in-person or virtually. Compared to the people without HDP who delivered during the same time period (n = 2307)	No difference in attendance between in-person and virtual groups at follow-up primary care visit in first year postpartum: 42% vs 38% (*P* = 0.70); nor cardiovascular risk factor screening (multiple risk factors evaluated). Significantly more attended primary care visit in those seen at clinic compared to overall population: 43% vs 30% (*P* < 0.01)
Soffer et al. 2023 [65]	Interventional Cohort Study	People with GDM in New York, U.S	74	Joint scheduling of 6-week postpartum appointment and 2-month infant check. Primary outcome: attendance at postpartum visit. Secondary outcomes: rates of glucose testing and infant visit attendance	No significant difference in attendance at 6-week review (69%) compared to historical controls (69%) (*P* = 0.84); 76.5% had glucose testing requested and 43.6% completed testing. Comparison between rates of glucose testing was not performed
Telehealth					
Hirshberg et al. 2018 [35]	Randomised Controlled Trial	People with HDP in Philadelphia, U.S	206	Participants received usual care with office-based blood pressure check, compared with intervention of text-based monitoring using a home blood pressure cuff and automated platform	Increase in blood pressure check in first 10 days postpartum from 44 to 92% (*P* < 0.001, adjusted odds ratio [OR] 58.2, 95% CI 16.2–208.1) No difference in attendance at 6-week postpartum visit (69% vs 58%, *P* = 0.11)
Hoppe et al. 2019 [66]	Interventional Cohort Study	People with HDP in Wisconsin, U.S	55	All participants received equipment and training to measure blood pressure and oxygen, and transmitted measurements to research team via tablet device. Telehealth visits and postpartum clinic appointment were scheduled	Feasibility study which found high satisfaction rates of using Telehealth post-partum. 52 of 55 participants completed the postpartum clinic visit at 6 weeks
Multi-approach programme					
Bose Brill 2021 [67]	Prospective Case–Control Study	Mother-infant dyads with GDM in Ohio, U.S	137	Concurrent mother and infant clinic appointments, with patient navigator assisting with childcare and transport issues, and supported by phone and electronic health record reminders	Increase in postpartum attendance from 58 to 95% (*P* < 0.001) and Type 2 diabetes screening (including OGTT, fasting glucose, random glucose and HbA1c) at 4–12 weeks postpartum from 79 to 87% (*P* < 0.001)
Vesco 2012 [68]	Retrospective Cohort	Participants with GDM in Oregon and Washington State, U.S	379	Staff received specialised training on GDM, handout was provided to people with GDM detailing follow-up testing and long-term risks, and electronic algorithm was developed which detected when postpartum testing had not been performed by 3 months; a nurse care manager then contacted these participants to remind them of testing recommendations	No change in postpartum testing from (54% vs 60%, *P* = 0.18) by 3 months A second reminder (of any type including telephone, letter or e-mail) increased testing at any time from 60 to 72% (*P* = 0.01)

*CI* confidence interval, *GDM* gestational diabetes mellitus, *HDP* hypertensive disorders of pregnancy, *OGTT* oral glucose tolerance test, *U.S.* United States

*Statistical analysis included where available in original papers

A limited number of strategies have been implemented in the antepartum period with the aim of improving postpartum testing rates. These include focused antepartum education [55], and use of care co-ordinators [46, 57], and in all studies have improved testing rates. Strategies implemented postpartum have been more widely trialled. Reminder systems (to people with GDM or HDP, their clinicians or both) have been examined with mixed outcomes [56, 60–63, 68]. Reminder systems where clinicians also received reminders, and which were implemented in the early postpartum period, appeared to achieve better testing rates.

Other successful approaches include the use of postpartum patient care co-ordinators and patient navigators. These roles may overlap: we define care co-ordination as a role that focuses on the provision of individualised care such as co-ordinating appointments and following up to ensure attendance, but which does not necessarily extend to in-person assistance; whereas patient navigators provide longitudinal care, often attending appointments with women, and provide a more culture-specific service. Both these strategies have been found to increase postpartum GDM testing [28] and early surveillance in people with chronic hepatitis B infection, another condition frequently diagnosed antepartum requiring long-term co-ordination of care [59].

Administrative assistance in booking testing and appointments has not been trialled extensively and understanding of the potential benefits of this approach remain limited. One study found improvement in testing rates in a small population with GDM when this approach was combined with patient incentives [58]. Specialist postpartum clinics have been trialled in those with HDP to facilitate transition to ongoing primary care follow-up, with financial viability and attendance rates of 85% demonstrated in one clinic providing intensive monitoring in the early postpartum period, including provision of home blood pressure monitoring resources [48]. Another multidisciplinary clinic providing both in-person and virtual care to women at a median of 11 weeks postpartum, had attendance rates of 80%, and included blood pressure testing for all attendees at the time of the visit, and lipid panel testing forms provided for metabolic screening [64]. Uptake of lipid testing for clinic attendees was low, at 53%, although no baseline rate was available for comparison [64]. No difference was identified in clinic attendance rates, nor postpartum lipid testing between those who attended virtually versus face to face [64]. Finally, models of care which integrate parent and infant postpartum visits into the same appointment have demonstrated variable success in improving rates of postpartum glucose testing [65, 67].

The use of Telehealth provides opportunities for flexible modalities of service delivery. As discussed, a hybrid clinic found no difference in either uptake of clinic use as measured by attendance rates, nor in metabolic screening, between participants who received virtual versus face to face care [64]. Use of remote monitoring of blood pressure has also been trialled for people with HDP, with successful outcomes in early testing [35] but not 6-week visit attendance [66]. Integration of technology as part of multi-approach programmes may enhance overall outcomes and is deserving of further attention.

## Tailoring strategies to optimise adherence to testing

Higher adherence to testing recommendations were seen with strategies which personalised healthcare, provided education and addressed individual barriers, such as through use of patient navigators, provision of environments which supported childcare and breastfeeding, and reduced time burden of testing [49]. This suggests that consumers are motivated to engage in healthcare when they are supported to do so according to their individual needs. Personalised healthcare, for example by use of a patient navigator to support individual needs, can also address a number of the barriers to accessing care, and care can be tailored in a way that allows equitable access.

Telehealth provides an opportunity to overcome the time and financial burden associated with travel and childcare, but introduces risk of further inequity via the digital divide if cost of technology and device access is placed on the individual [69]. For women who do not speak English, the logistics of ensuring adequate access to interpreters within the telehealth setting can add another layer of complexity. Furthermore, there may be unintended consequences of telehealth appointments including missed opportunities for screening, vaccination and physical examination. Care must be taken when designing and evaluating initiatives to ensure that unintended consequences that widen gaps in healthcare access do not occur.

Limited qualitative data exists on what consumers of postpartum care want and need after diagnosis of high-risk conditions to enable them to access recommended testing. A cross-sectional telephone survey of people with GDM in Australia explored the barriers to post-partum screening in detail [49]. Logistic challenges such as the time burden of doing a two-hour OGTT and childcare access featured prominently. These concerns were echoed in an earlier study which also identified that many participants either perceived they were ‘healthy’ or were fearful about receiving a diagnosis of type 2 diabetes after postpartum glucose testing [70]. With any programme designed to improve engagement in healthcare, it is essential to understand the needs and wishes of the intended consumers. Involvement of consumers in the developmental stages of such programmes, and feedback from those who receive care as part of an evaluation process, are recommended.

## Key findings

The postpartum period, or the ‘fourth trimester’, provides a unique healthcare opportunity, when otherwise mostly healthy people access healthcare regularly. Many pregnant people will receive an antepartum diagnosis associated with potential longer-term adverse health outcomes. Primary prevention can be implemented to reduce these risks and/or delay onset. However, this must be provided equitably and address disparities in healthcare access within the highest risk populations.

Despite several different strategies trialled in a wide variety of populations, there is not conclusive data on the best method to improve attendance at postpartum appointments and to engage those with high-risk conditions in longer-term follow-up.

Strategies that have shown promise include: *focused antepartum education*, for example with a diabetes educator [55], nutritionist, or with education booklets [57]; *use of patient care co-ordinators* providing assistance with booking testing appointments and following up those who did not attend to arrange in-home testing [28, 46]; and *patient navigators* providing individualised assistance with education, co-ordination of testing, and assistance to attend [59].

In addition, creating environments for postpartum testing which support the unique needs of parents with young infants, such as provision of breastfeeding-friendly spaces with options for childcare, may help reduce inequities in access [49]. Systems which facilitate remote testing opportunities, for example blood pressure measurements, can help reduce geographical and transport barriers. The expanding field of telemedicine has yet to be fully realised in this area of healthcare but has potential to enhance systemic provision of care.

## Avenues for future research

Several gaps have been identified requiring further research. Firstly, the data around both rates of postpartum follow-up at the routine postpartum check and uptake of recommended testing in those at higher risk of long-term conditions is limited to a small number of geographical areas and does not capture national rates. Estimations of the scale of these issues are essential to healthcare and resource planning.

Secondly, careful thought is required about how to provide effective, scalable strategies to support people at this uniquely challenging time, and which provide an opportunity to empower them in managing their immediate and longer-term health. Thirdly, many programmes employ nursing and allied health practitioners such as social workers and administrative staff to provide education and care, with most of these based within the hospital system. Integration with existing community services beyond primary care practitioners may help broaden access, such as through community pharmacists and pathology testing centres.

Further, the role of telemedicine requires further exploration particularly in settings with geographical barriers to care and for those with challenges to in-person attendance such as childcare needs. The potential for remote monitoring, for example with blood pressure and blood glucose level testing, are areas of promise.

Finally, the needs of populations with specific health, cultural and equity imbalances must be reviewed in depth, and consumers from these groups included in co-design and evaluation of strategies.

## Quick points

- Postpartum pathways of care are poorly defined and attendance at recommended visits is often suboptimal
- Uptake of recommended testing after high-risk conditions including gestational diabetes and hypertensive disorders of pregnancy is low
- Barriers to uptake of testing include individual, sociodemographic and systemic factors which often overlap with drivers of inequity in access to healthcare in general
- Strategies to improve adherence to recommended testing include specialist postpartum clinics, patient education and the use of patient navigators
- Further research into engagement with healthcare, and optimal strategies to support postpartum populations including through a culturally sensitive and equitable lens are urgently required
